# Stage-specific expression of the mitochondrial co-chaperonin of Leishmania donovani, CPN10

**DOI:** 10.1186/1475-9292-4-3

**Published:** 2005-04-29

**Authors:** Fanny Beatriz Zamora-Veyl, Manfred Kroemer, Dorothea Zander, Joachim Clos

**Affiliations:** 1 Bernhard Nocht Institute for Tropical Medicine, Bernhard Nocht St. 74, D-20359 Hamburg, Germany

## Abstract

**Background:**

Leishmania spp., in the course of their parasitic life cycle, encounter two vastly different environments: the gut of sandflies and the phagosomes of mammalian macrophages. During transmission into a mammal, the parasites are exposed to increased ambient temperature as well as to different carbon sources. Molecular chaperones or heat shock proteins are implicated in the necessary adaptations which involve the ordered differentiation from the flagellated, extracellular promastigote to the intracellular amastigote stage.

**Results:**

Here, we show that the Leishmania donovani co-chaperonin, CPN10, is synthesised to a significantly increased concentration during in vitro differentiation to the amastigote stage. We show by fluorescence microscopy and by immunogold electron microscopy that, like its putative complex partner CPN60.2, CPN10 is localised to the single, tubular mitochondrion of the parasites and, moreover, that it co-precipitates with CPN60.2, the major mitochondrial chaperonin of Leishmania spp..

**Conclusion:**

Our data indicate an increased requirement for CPN10 in the context of mitochondrial protein folding during or early in the mammalian stage of this pathogen. Moreover, they confirm the CPN60.2 as bona fide mitochondrial GroEL homologue in L. donovani and the postulated interaction of eukaryotic chaperonins, CPN60 and CPN10.

## Background

The euglenozoa which, beside the euglenophyta, also include the pathogenic protozoa of the Order Kinetoplastida, have branched off early in eukaryotic evolution. The kinetoplastida have developed, or retained, molecular mechanisms not shared by most Crown Group Eukaryota, such as trans-splicing of messenger RNA, RNA editing, and polycistronic transcription. The genetic information of their single mitochondrion, the so-called kDNA, is localised to the kinetoplast, an organelle unique to this order. The kinetoplast is part of a tubular, single mitochondrion that shows little morphological likeness to the mitochondria of Crown Group Eukaryota. The kinetoplastida encompass two genera of parasitic pathogens, Trypanosoma and Leishmania, both of which responsible for considerable human morbidity and mortality.

The parasites of the genus Leishmania exist in two morphologically distinct life cycle stages. The slender, flagellated, promastigote proliferates in the alimentary tract of female sandflies at ambient temperature. The amastigote, a rounded, intracellular form, with no protruding flagellum, persists in the phagosomes of mammalian macrophages and at temperatures of up to 39°C. The difference of temperatures in the two environments not only triggers the respective stage development [1], but also poses a challenge to the system of protein chaperones. At least two heat shock proteins, Hsp100 and CPN60.2, show significantly induced abundance during the amastigote stage [2-4].

Apart from the temperature difference, the two environments, insect gut and macrophage phagosome, also offer different qualitative and quantitative composition of carbon sources. The life cycle stage differentiation, therefore, must include an adaptation of the parasites' metabolic pathways. Subcellular compartments, e.g. the single tubular mitochondrion, also have to undergo adaptation. The folding of a different set of metabolic enzymes after their translocation across the outer and inner mitochondrial membranes can be expected to challenge the mitochondrial chaperone system.

Import of nuclear encoded proteins is vital for the function of mitochondria. A complex machinery of chaperone proteins is required for the unfolding of mitochondrial proteins, their trans-membrane transport, and their refolding inside the mitochondrial matrix [5]. The latter is thought to be brought about by the 60 kDa chaperonin (CPN60). This protein is a functional and structural homologue to the GroEL chaperonin of the eubacteriae. The function of GroEL, being the subject of considerable research efforts, is well understood [6,7]. In bacteria, GroEL forms barrel-like structures of two rings of 7 subunits each, with two central cavities. 7 subunits of the GroES co-chaperonin form a lid that covers one of the cavities. Folding intermediates of client proteins are bound inside one cavity where they may attain energetically favoured conformations without interference from other polypeptides.

Less is known, by comparison, about the function of eukaryotic GroEL homologues, the CPN60 proteins. They are nuclear-encoded mitochondrial proteins. Rather than forming two rings of seven GroEL subunits, the chaperonin complex in mitochondria consists of a single ring of seven CPN60 and one ring of CPN10 subunits, and it was shown that the function of CPN60/CPN10 complexes inside the mitochondria is similar to the chaperonin function of bacterial, cytosolic GroEL/GroES complexes [8,9]. GroES may also play a role in modulating the substrate specificity of GroEL. For instance it was reported that GroES binding shifts the preference of GroEL for hydrophobic amino acids towards amino acids with hydrophilic side chains [10]. In yeast, not all CPN60 substrates need CPN10 for proper folding. Other proteins require both CPN60 and CPN10, while newly imported CPN60 depends on CPN10 for correct folding [11].

We had previously identified the Leishmania donovani CPN60.2 gene which encodes the mitochondrial 60 kD chaperonin [4]. This protein shows increased expression during in vitro promastigote to amastigote differentiation, indicating an altered requirement for CPN60 in the amastigote stage. A second, diverged gene copy, CPN60.1, is not expressed to detectable levels in any in vitro culture stage of L. donovani or L. major [4]. To better understand mitochondrial protein chaperoning during the leishmanial life cycle, we identified the CPN10 gene of Leishmania donovani, determined its abundance in the two life cycle stages, and analysed the subcellular localisation of the gene product and its association with CPN60.2.

## Results

### Amplification of L. donovani CPN10 DNA

An alignment of the known eukaryotic CPN10 amino acid sequences revealed four limited stretches of sequence conservation (not shown). Oligonucleotides CPN10.1 to CPN10.4 were designed according to the codon bias of Leishmania spp.. Using L. donovani genomic DNA as template, combinations of the aforementioned primers were tested in a polymerase chain reaction. Only the combination of primers CPN10.1 and CPN10.4 yielded an amplification product of the expected size (not shown). The amplification product was then subcloned into the vector pBluescript and subjected to sequence analysis using standard pBluescript sequencing primers. The sequence analysis confirmed that the amplification product harboured probable CPN10 sequences (not shown).

### Isolation of a CPN10 genomic DNA clone

**Figure 1:**
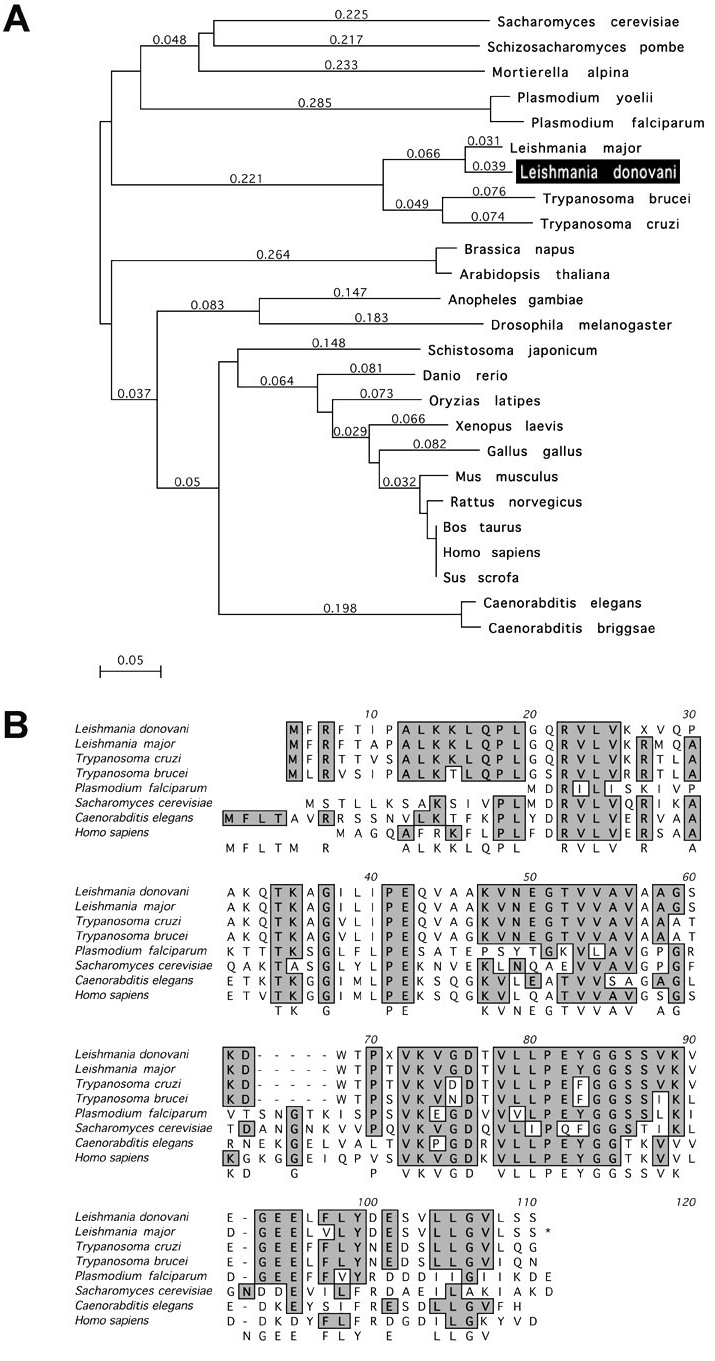
(A) Phylogenetic analysis (clustalW) of eukaryotic CPN10 family members. The bar shows the scale for sequence divergence. (B) Amino acid sequence alignment of the LdCPN10 and selected eukaryotic CPN10 family members. The alignment was perfomed using the clustalW algorithm.

The amplified putative CPN10 gene fragment was used to screen a L. donovani genomic DNA cosmid library [3]. Positive cosmids were subjected to a restriction digest and Southern Blot analysis to identify common subfragments which harboured the CPN10 gene(s). A 1.5 kb XhoI I fragment which hybridised with the CPN10 probe was subcloned into pBluescript and subjected to sequence analysis. The sequence (GenBank AF394959) contains an uninterrupted open reading frame of 303 bp encoding a putative 10.7 kDa polypeptide. A sequence comparison by BLAST search confirmed this putative polypeptide as related to eukaryotic and prokaryotic co-chaperonins. Figure 1A shows the position of the L. donovani CPN10 in the lineage of eukaryotic CPN10 proteins. L. donovani CPN10 groups within the kinetoplastid homologs. Figure 1B shows an alignment with selected eukaryotic CPN10 polypeptides. The high degree of sequence conservation between LdCPN10 and its counterparts in higher eukaryotes indicates a conserved function.

### Southern Analysis

**Figure 2:**
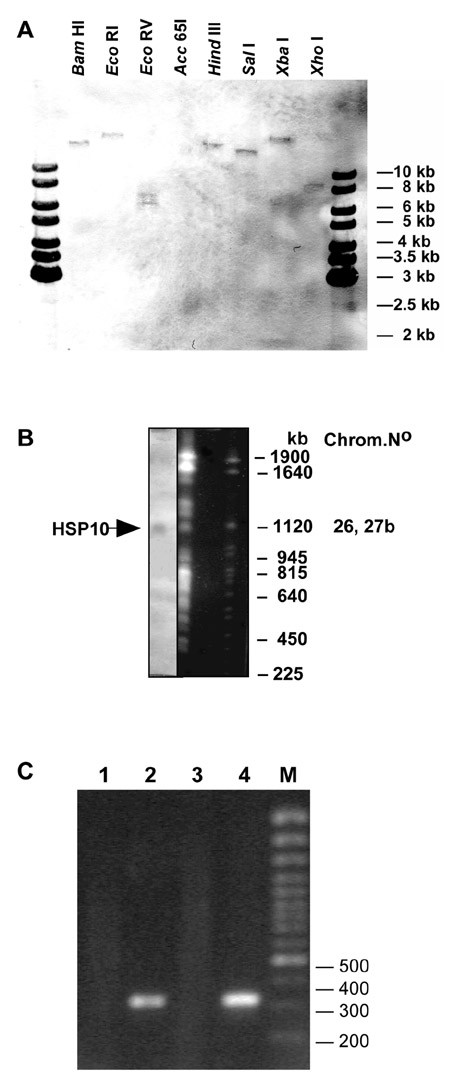
(A) Southern analysis of L. donovani genomic DNA. The DNA (2 μg) was digested with restriction enzymes as indicated and separated on a 1 % agarose gel. After Southern transfer the membrane was probed with a CPN10 specific probe. The positions of size markers (1 kb ladder, Fermentas) are indicated. (B) Pulsed field gel electrophoresis of L. donovani chromosomes. The gel was stained with ethidium bromide and photographed (right). After denaturation the DNA was blotted on a Nylon membrane and probed with a CPN10 specific probe (left). The sizes of S. cerevisiae chromosomes which were run alongside as size markers are shown to the right. The chromosome band which hybridised with the CPN10 probe is marked by an arrow. (C) RT-PCR of L. donovani RNA. Whole cell RNA was isolated from L. donovani cells and subjected to reverse transcription at 37°C for 60 min using an oligo-dT primer. An aliquot of the cDNA reaction mix was inactivated at 65°C for 10 min prior to incubation at 37°C. Either RNA (lane 1), the cDNA (lane 2), the heat inactivated cDNA mix (lane 3) or genomic DNA (lane 4) were added to a PCR reaction mix using CPN10 specific primers. The sizes of selected marker DNA molecules is shown to the right.

Within the haploid set of chromosomes, L. donovani CPN10 appears to be single copy. This was determined by Southern Blot analysis of L. donovani genomic DNA, digested with 7 restriction endonucleases (Figure 2A). The pattern of restriction fragments does not indicate the presence of additional gene copies. We also performed pulsed field gel electrophoresis (Figure 2B) to determine the chromosomal localisation of the CPN10 gene. The CPN10 specific probe hybridised to a chromosomal band of approximately 1,100 kb. This size corresponds to chromosomes 26 or 27b. This was confirmed when sequencing of the L. major genome was finished; two copies of CPN10 are found on chromosome 26 .

### RT-PCR analysis

Northern analysis of L. donovani RNA using a CPN10-specific probe did not yield any bands in the expected size range. To ascertain that the putative CPN10 gene encodes a stable RNA, we performed RT-PCR. Whole cell RNA was reversely transcribed using an oligo-dT primer. The first strand cDNA was used as template in a PCR amplification using primers CPN10-1 and CPN10-4. Negative controls included an amplification of material from a cDNA synthesis mix that had been inactivated at 65°C for 10 min (Figure 2C, lane 1), and an amplification with an equivalent amount of whole cell RNA as template (lane 3), to exclude DNA contaminations in the RNA preparation. Both reactions did not give rise to an amplification product. The reaction with cDNA as template (lane 2), however, produced a ~350 bp PCR product, much the same as the reaction with genomic DNA as template (lane 4). We conclude that stable, polyadenylated RNA is produced from the CPN10 gene.

### Production of anti-CPN10 antibodies

**Figure 3:**
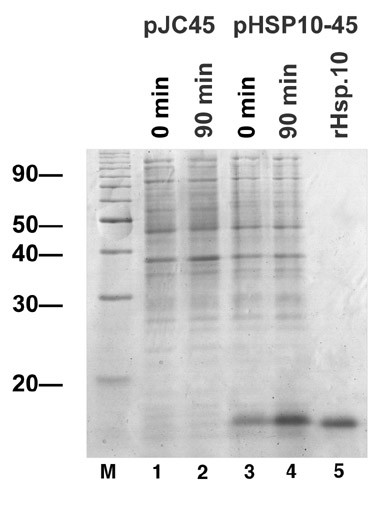
Expression of L. donovani CPN10 in E. coli. The plasmids pJC45 or pCPN10-45 was used to transform E. coli strain BL21 (DE3) [pAPlacIQ]. Expression was induced by addition of IPTG, and the bacteria were harvested. After lysis, aliquots of the cultures before and after IPTG induction were analysed by SDS-PAGE and Coomassie Blue staining. The lysate containing the rCPN10 was subjected to metal chelate chromatography to purify the rCPN10 by virtue of its histidine tag. The purified rCPN10 was also subjected to SDS-PAGE analysis.

The CPN10 open reading frame was subcloned into pJC45, a derivative of pJC40 [4,12], for expression in E. coli strain Bl21 (DE3) [13]. His-tagged CPN10 was purified by metal chelate chromatography (Figure 3) and used to immunise laying hens. Antibodies were prepared from egg yolk before and after immunisation to yield pre-immune and CPN10-specific antibody preparations. In Western Blot analyses, these antibodies faithfully recognised recombinant rCPN10 (not shown).

### LdCPN10 expression is induced in the amastigote

**Figure 4:**
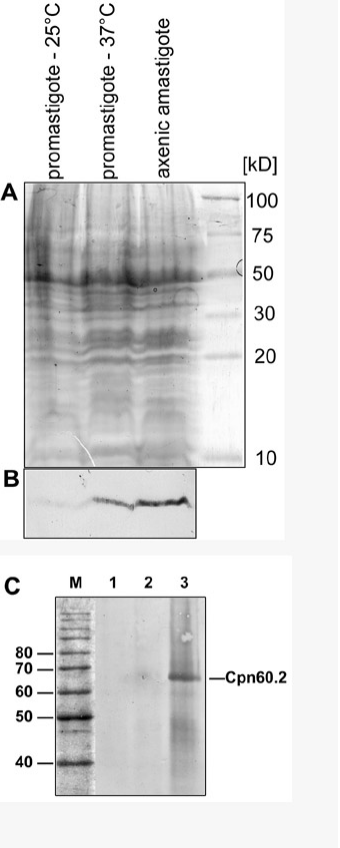
(A) SDS-PAGE of various in vitro culture forms of L. donovani. Equivalent protein aliquots from L. donovani promastigotes, cultivated at either 25°C (promastigote -25°), at 37°C (promastigote -37°), and from axenic amastigotes after 3 days of in vitro differentiaton were subjected to Tricine Gel Electrophoresis and to SDS-PAGE. Protein bands were visualised by Coomassie Brilliant Blue staining. (B) Immunoblot analysis. A parallel gel was subjected to Western transfer and probed with an anti-CPN10 antibody. (C) Co-immune precipitation. Leishmania donovani lysate was precipitated using anti-CPN10 antibodies, separated by SDS-PAGE and subjected to WesternBlot analysis with anti-CPN60.2 antibodies. Lanes 1 and 2 are negative controls representing immune precipitations without anti-CPN10 antibody and with a preimmune antibody preparation, respectively. Lane 3 represents the CPN10 immune precipitation proper. The position of CPN60.2 is indicated.

Using the anti-CPN10 antibodies, we performed immunoblot analysis on lysates of L. donovani promastigotes before and after heat shock, and of axenically cultured amastigotes (Figure 4B). In promastigotes cultured at 25°C, we observe only a faint band in the expected size range. After a 24 h treatment of the promastigotes at 37°C, a signal is observed. The CPN10 signal increases strongly during in vitro amastigote differentiation. Figure 4A shows identical samples separated by SDS-PAGE and Coomassie Blue staining. We conclude that Leishmania donovani CPN10 is expressed preferentially in the amastigote stage, induced at least in part by the elevated temperature.

### CPN10 is the co-chaperonin in L. donovani

By analogy, CPN10 is expected to act as co-chaperonin to the 60 kDa chaperonin, CPN60. However, there are two diverged members of the CPN60 gene family in L. donovani, CPN60.1 and CPN60.2. Of these, only CPN60.2 could be shown to encode a protein which localised to the mitochondrion [4]. We therefore tested whether CPN10 interacts with CPN60.2 protein in L. donovani and performed co-immune precipitation. L. donovani cell lysates were incubated with anti-CPN10 antibodies, anti-chicken IgG (rabbit) and Protein-A agarose. The precipitated material was then analysed by immunoblot using anti-CPN60.2 antibody. As shown in Figure 4C, the anti-CPN10 precipitate contains a protein which reacts with the anti-CPN60.2 antibodies. We conclude that CPN60.2 is the bona fide chaperonin and that CPN10 serves as its co-chaperonin.

### Subcellular localisation of LdCPN10

**Figure 5:**
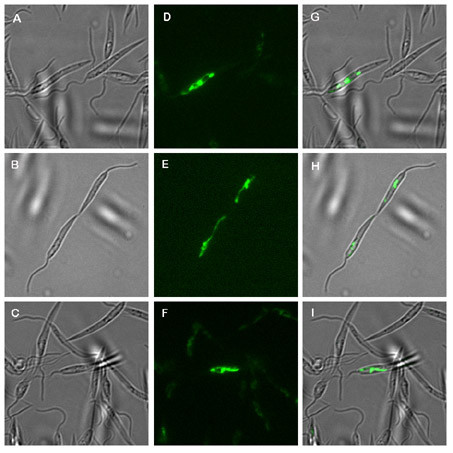
Fluorescence microscopy. L. donovani promastigotes transfected with a CPN10/GFP gene chimera on plasmid pIRCPN10::GFP were subjected to bright field microscopy at 63× magnification (panels A-C). The same microscopic fields were viewed under UV excitation at XXX nm (panels D-F). Panels G-I show the overlays.

Since CPN10 serves as co-chaperone to CPN60 we expected CPN10 to localise to the kinetoplast/mitochondrion complex inside the leishmaniae. To test this prediction, the CPN10 coding sequence was fused to a GFP (Green Fluorescent Protein) coding DNA in the expression vector pIRmcs3-. The linearised expression construct was recombined into the rRNA locus of L. donovani to yield stable expression of the CPN10::GFP chimera. The recombinant parasites were cultivated as promastigotes and analysed by fluorescence microscopy. The results are shown in Figure 5. We observe brightly fluorescent tubular structures in the promastigotes. No fluorescence is observed in the cytoplasm. This indicates that the CPN10 sequence can quantitatively direct GFP into a subcellular compartment or organelle.

**Figure 6:**
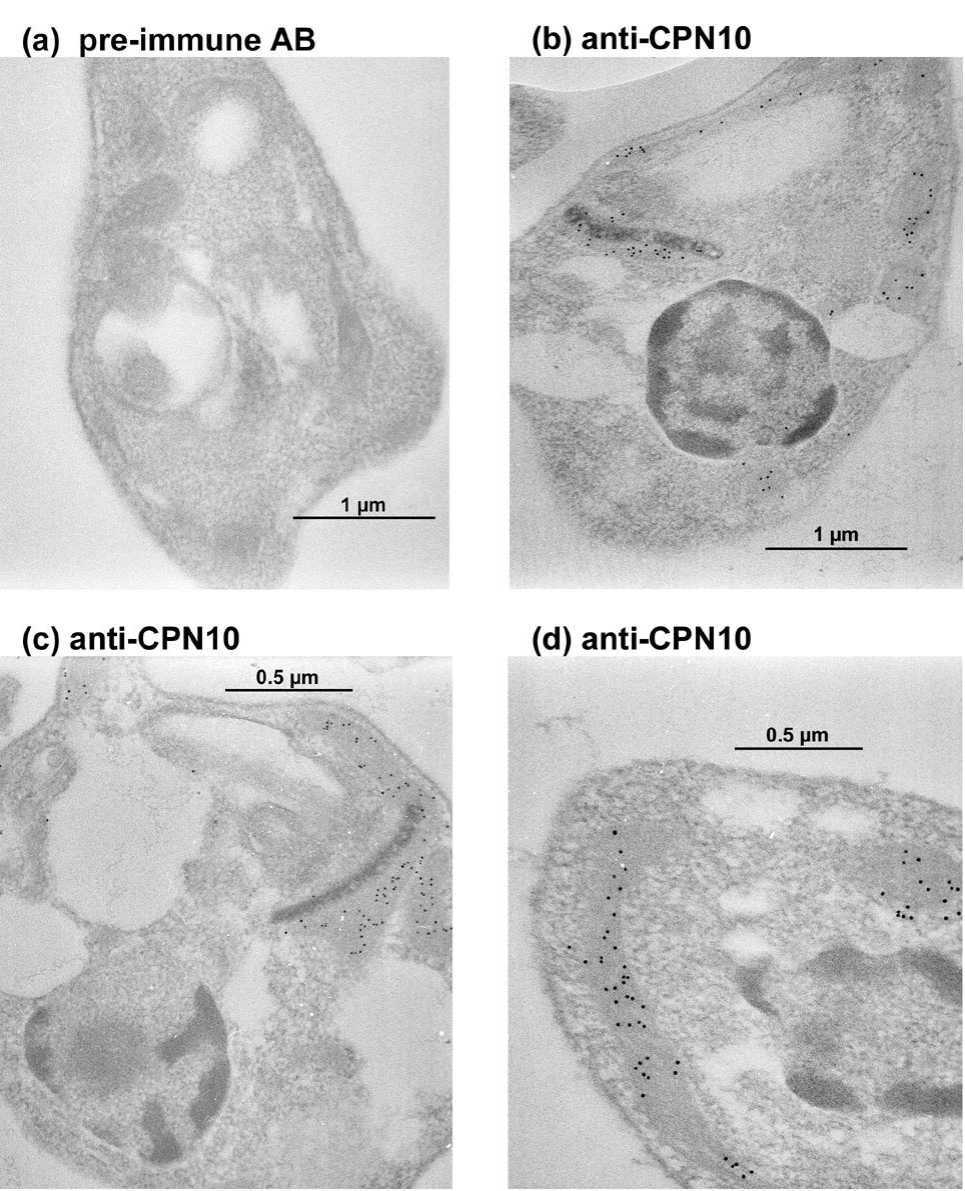
Immunogold electron microscopy. Heat-shocked promastigotes (a,d) or axenic amastigotes (b,c) were embedded in LR-White. Microsections were stained with pre-immune antibodies (a) or anti-CPN10 (b-c) antibodies. Antibody binding was detected using rabbit anti-chicken IgG and Protein A Gold (10 nm). Annotations: n = nucleus; k = kinetoplast; m = tubular mitochondrium.

To identify the subcellular compartment that harbours CPN10, we performed immune electron microscopy using sections of heat stressed promastigotes and of axenic amastigotes. Figure 6 shows the result. We find the gold particles associated both with tubular structures and with the kinetoplast. We conclude that CPN10 is a mitochondrial protein.

**Figure 7:**
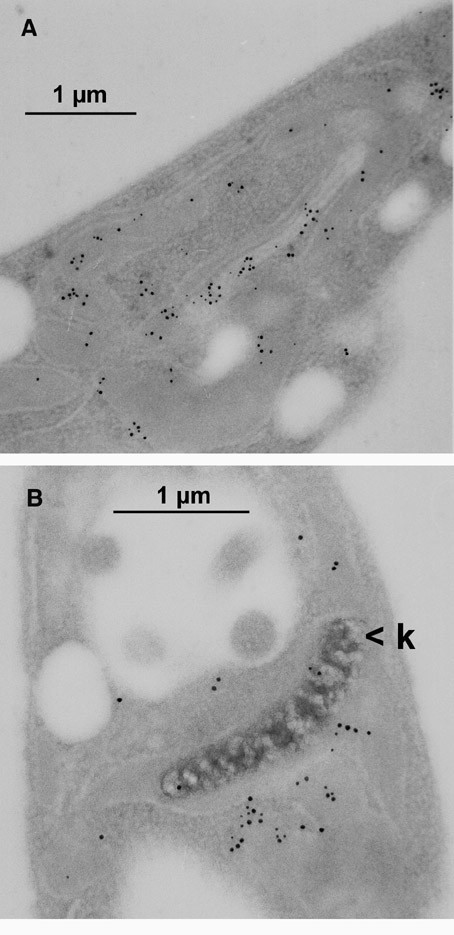
Double immune electron microscopy. Leishmania cells that express CPN10::GFP chimera were embedded and microsections were prepared. After staining with anti-GFP Mab and anti-mouse immunogold (5 nm), sections were treated with anti-CPN60 antibodies, rabbit anti-chicken IgG, and Protein A gold (10 nm). Thus, 10 nm gold particles represent CPN60 and 5 nm gold particles represent CPN10::GFP. k = kinetoplast.

When we analysed the subcellular localisation of CPN60.2 in L. donovani, this chaperonin, too, localised to a tubular subcellular compartment. However, we did not find it associated with the kinetoplast. Therefore, the exact subcellular localisation was still unclear. Using the strain which overexpressed the CPN10::GFP chimera, we performed a co-immune electron microscopy. We used anti-GFP mAB in conjunction with anti-mouse immunogold particles (5 nm). Then the sections were co-stained using anti-CPN60 antibody, anti-chicken IgG (rabbit), and protein A gold (10 nm). As shown in Figure 7, CPN10::GFP and CPN60 colocalise to the same tubular compartment. This shows that CPN60.2 is indeed a mitochondrial protein in Leishmania.

## Discussion

We have cloned and analysed the CPN10 gene of the protozoan parasite Leishmania donovani. This is, to our knowledge, the first characterisation of a protozoan member of the conserved GroES family of co-chaperonins. Since CPN10 is a very small protein with an impact on mitochondrial function, the restraints on sequence variation should be rather tight. Accordingly, Leishmania CPN10 is >60 % similar to mammalian CPN10 sequences. A comparison of the L. donovani CPN10 gene with other eukaryotic GroES homologs further confirms that the leishmaniae belong to a remote entity among the eukaryota, clearly separated from the Eukaryotic Crown Group. A similarly diverged position was observed for the GroEL homologue of L. donovani, CPN60.2 [4].

The gene for CPN10 is single copy in the L. donovani genome. This is in contrast to the CPN60.2 gene which is present in two copies, in addition to a, as far as known silent, CPN60.1 gene [4]. It is also in contrast with the situation in L. major. According to the L. major genome database, the L. major CPN10 is encoded by two gene copies located in tandem. If confirmed, this difference could serve to distinguish both Leishmania species which have overlapping endemic regions.

Eukaryotic CPN10, like its bacterial counterpart, GroES, is assumed to act as co-chaperonin to CPN60 in the mitochondrial matrix. Immune electron microscopy reveals that CPN10 localises both to the kinetoplast and to the tubular part of mitochondrion. More importantly, co-immunoprecipitation experiments establish a direct interaction between CPN10 and CPN60.2. This not only indicates a function of CPN10 as bona fide co-chaperonin, but also establishes CPN60.2 as the Leishmania GroEL homolog. In addition, the mitochondrial localisation of both proteins is now well established.

The mitochondrial transport signal sequence of CPN10 can direct the import of the much larger CPN10::GFP chimera into the mitochondria and the kinetoplast. The chimera is expressed stably from the integration site within the rRNA gene repeats. The chimera may serve for future experimental designs as mitochondrial localiasation marker and as marker for the integrity of the mitochondrion.

The CPN10 gene is expressed preferentially under heat stress and in axenic amastigotes of L. donovani. The putative complex partner, CPN60.2, also shows increased abundance under heat stress [4]. The induction of CPN10, however, is more pronounced, and comparable to the induction of Hsp100 [3]. In this regard, three heat shock proteins, Hsp100, Cpn60.2, and CPN10, differ from the major chaperones, Hsp70 and Hsp90 (Hsp83). The latter show high constitutive abundance and only a marginal increase during heat stress and in the amastigote stage [3,14]. It is likely that these different expression kinetics reflect different roles during the life cycle. Hsp100 is required only in intracellular amastigotes of L. donovani and has a role in amastigote specific gene expression [15]. CPN60.2, and even more so, CPN10, are obviously also in increased demand during the amastigote stage.

Is there a specific function for CPN10 during the mammalian stage of the parasite's life cycle? It has been suggested that GroES can alter the affinity of GroEL for amino acid side chains [10]. In the yeast, substrate proteins may fold either independently of CPN10 or in a CPN10-dependent manner [11]. This raises the possibility that the preferential expression of CPN10 in amastigotes of L. donovani may reflect a change in the substrate specificity of the mitochondrial chaperonin complex, necessitated by the change of environment and the concomitant changes in nutrient availability. Since CPN10 is a single copy gene that is hardly expressed in the highly proliferative promastigote stage, it may be a suitable target for a gene replacement approach. This should allow to assess the role of CPN10 for the virulence and pathogenicity of Leishmania parasites.

## Conclusion

We have identified the Leishmania donovani homologue of the bacterial co-chaperonin, GroES, and assigned the name CPN10 to the gene and the protein. CPN10 is a heat shock protein and shows increased intracellular concentration at an elevated temperature and in axenically cultivated amastigotes of L. donovani. CPN10 localises to the mitochodrial compartment and co-localises with the GroES homologue, CPN60.2. A direct interaction of both proteins was demonstrated using co-immuno precipitation. Both CPN10 and CPN60.2 are therefore suitable markers for the mitochondria of Leishmania spp. and related species. Moreover, the increased concentration in the amastigote stage suggests a role of CPN10 in this stage.

## Methods

### Parasite strains and culture

For our analyses we used the Lo8 strain of L. donovani, a gift from D. Zilberstein. Promastigotes were routinely cultivated at 25°C in supplemented M199 medium [3]. Cell density was monitored using a Schaerfe Systems CASY Cell Counter. In vitro promastigote to amastigote differentiation was performed as described [3].

### PCR amplification of CPN10 DNA

Four primers were delineated from an amino acid sequence comparison of known GroES and CPN10 proteins. The extreme bias in Leishmania towards G and C residues in the wobble positions of codons was used to create primers with minimal degeneracy:

primer CPN10.1: CCGCTGTTCG ACCGCGTGCT GG

primer CPN10.2: GGCGGCATCR TGCTGCC

primer CPN10.3: CGGCAGCAYG ATGCCGCC

primer CPN10.4: CAGCACCTTG TCGCCCACCT TCAC

100 ng of L. donovani genomic DNA was mixed with 40 ng of oligonucleotide primer pairs, 25 nmoles of each dNTP, 10 × reaction buffer, and 1 unit of Taq DNA polymerase (Beckman) in a 50 μl reaction. The reaction mix was incubated for 1 min at 95°C, 1 min at 55°C, and 1 min at 72°C. The cycle was repeated 35 times. 10% of the reaction was analysed on a 1% agarose gel. Negative controls included each primer by itself and omission of template DNA. Bands absent from the negative controls were recovered by preparative agarose gel electrophoresis and by using glass beads adsorption (PureGen Kit, Biozyme). PCR products were subcloned using the TA cloning kit (Invitrogen) and subjected to sequence analysis..

### Reverse transcriptase (RT-) PCR

Total RNA from promastigotes stage parasites strain Lo8 was prepared using a Ribolyser Kit (Hybaid). cDNA synthesis was carried out using the First Strand synthesis kit (Pharmacia) with the enclosed (dT)_18 _primer and 5 μg of total RNA. The cDNA was then amplified enzymatically using the primers CPN10.1 and CPN10.4 The amplification was carried out as described above.

### Library screening

We used a cosmid library of L. donovani previously described in [16]. The library was screened by hybridisation with digoxigenin-labeled DNA probes derived from the subcloned amplification products. Hybridisation was performed at 65°C in HYB 9 solution (Biozyme) for 16 h. Washes were performed at decreasing SSC concentrations (6 × to 0.2 × SSC, 0.5 % SDS) at 65°C. Filters were developed using anti-digoxigenin FAB/AP conjugate (Boehringer Mannheim) followed by colorimetric staining with NBT/BCIP. Positive cosmid clones were picked, rescreened, and verified in an amplification reaction using CPN10-specific primers.

### Subcloning and sequence analysis

Cosmid DNA from positive clones was subjected to restriction endonucleased digest and Southern Blot to determine suitable restriction endonucleases. Cosmid DNA was then digested at a preparative scale and subcloned into an appropriately cleaved pBluescript KS+ vector (Stratagene) or pJC45 vector [4]. Plasmid subclones were then subjected to bidirectional primer walking sequence analysis, starting from the known sequences of the PCR amplificates. Sequencing was performed on an Applied Biosystems Model 370 sequencer using the DyeTerminator Kit.

### Sequence evaluation

Contig alignment, in silico translation, and sequence alignment were performed using the MacMolly Tetris and the MacVector software packages. Phylogenetic analyses were performed using the clustalW algorithm included with MacVector at default settings.

### Recombinant Expression of CPN10

The putative open reading frame of CPN10 was amplified using specific primers that modify the start codon sequence into a NdeI site and the stop codon sequence into an EcoRI site. The amplification products were cleaved with NdeI and EcoRI and ligated into the expression vector pJC45 [4], between the NdeI site and the EcoRI site. The expression plasmids were transformed into the bacterial strain BL21 (DE3) [pAPlacIQ], a gift from Olivier Payet, CNRS Toulouse. Bacteria were grown in CircleGrow medium (BIO 101) supplemented with 50 μg/ml Ampicillin and 10 μg/ml Kanamycin to OD600 = 0.5. IPTG was added to 1 mM and incubation was continued for 1 h. The purification of His-tagged proteins from bacterial lysates by metal chelate chromatography has been described [12,14].

To express CPN10::GFP chimera, the CPN10 coding sequence, minus the stop codon, was enzymatically amplified to create BamHI and HindIII sites at the 5' and 3' ends, respectively. Digested with BamHI and HindIII, the amplification products were ligated between the BamHI and HindIII sites of the yeast plasmid p426/PQ25 [17]. By this procedure, CPN10 and GFP coding sequences were fused in frame. The chimeric CPN10::GFP coding sequence was then excised with BamHI and XhoI and ligated into pIRmcs3- [18]. The plasmid pIRCPN10::GFP and the parent plasmid pIRmcs3- were linearised using SwaI and transfected into L. donovani by electroporation. Recombinant parasites were placed under ClonNAT (Werner Bioreagents) selection (100 μg/ml) and analysed by fluorescence microscopy.

### Immunisation and antibody preparation

The immunisation of laying hens and the preparation of antibodies from egg yolk has been described [19].

### Immunoblot analysis

SDS-PAGE and Western Transfer were performed as described [14,19]. Briefly, membranes were treated with blocking buffer (4% skim milk powder and 0.1% Tween 20 in DPBS), with antibodies at appropriate dilutions in blocking buffer, and with secondary antibody/alkaline phosphatase conjugate (Dianova) in blocking buffer. Blots were stained with nitrobluetetrazolium chloride and 5-bromo-4-chloro-3-indolyl phosphate.

### Immune precipitation

1 × 10^8 ^promastigotes were harvested by centrifugation and lysed in 500 μl solubilising buffer (50 mM Tris-HCl pH 7.5, 150 mM NaCl, 1% NP-40, and 2.5 μg × ml-1 each of aprotinin, leupeptin, pepstatin, and antipain). Lysates were centrifuged at 13000 × g, 4°C. Soluble proteins were incubated with 20 μl anti-CPN10 antibody for 1 h at 4°C. 350 μl of protein A-sepharose slurry were preincubated with anti-chicken IgG from rabbit and added. The slurry was further incubated at 4°C for 2 h. The immunoabsorbent was centrifuged and washed three times in washing buffer A (10 mM Tris-HCl pH 7.5, 150 mM NaCl, 0,2% NP-40, 2 mM EDTA), twice in washing buffer B (as above, except NaCl is 500 mM) and once in washing buffer C (10 mM Tris-HCl pH 7.5). For analysis on SDS-PAGE 250 μl of SDS sample buffer was added and the samples were heated for 5 minutes at 95°C. After centrifugation, 50 μl of the supernatant was loaded onto a 12% PA gel.

### Immune electron microscopy

Immune electron microscopy was performed essentially as described [3]. LR-White embedded microsections were treated with chicken antibody at 1:500 (anti-CPN10) or 1:2000 (anti-CPN60.2) in PBS (0.1 % Tween 20, 1% BSA). Anti-chicken IgG (rabbit) and Protein-A immunogold particles (10 nm) were used for detection. For detection of CPN10::GFP chimera, anti-GFP monoclonal antibody and 5 nm anti-mouse immunogold conjugate (Sigma) were used.

### Fluorescence microscopy

L. donovani promastigotes expressing CPN10:GFP chimera were grown to 2 × 10^7 ^cells ml^-1^. 1 ml of parasite culture was subjected to centrifugation for 10 min at 1500 × g. The supernatant was discarded and the cell pellet was resuspended in 200 μl of Dulbecco's PBS. 2 μl was applied to a multiwell microscope slide and subjected to fluorescence microscopy at 63-fold magnification. Samples were analysed in bright field and in the FITC channel using a Hitachi Model monochrome CCD camera. Images were taken and merged using the Improvision Open Lab^® ^software package and exported in TIFF format. Cropping and juxtapositioning were done using Adobe Photoshop^® ^software.

### Pulsed Field Gel Electrophoresis

Leishmania cells were harvested by centrifugation and washed twice in PBS. Following centrifugation, parasites were resuspended in PBS and mixed with an equal volume of prewarmed 1,5 InCert agarose (FMC BioProducts, Rockland). This mixture was aliquoted in block formers (2 × 107 parasites per block, Pharmacia). To lyse parasites, the agarose blocks were incubated in 2 mg/ml proteinase K (1% Laurylsarkosyl, 0,5 m EDTA pH 9.0) at 37°C for 48 h, and stored in 0.5 EDTA at 4°C.

PFGE was performed at 13°C in 0,25 × TBE buffer under the following conditions: intervall in sec: 100-10 log; switching angle: -110 lin; voltage: 200-150 log (Rotaphor, Biometra).

Saccharomyces cerevisiae strain YPH80 chromosomes (New England Biolabs) were used as size standards.

After staining with ethidium bromide (1 μg ml-1) and strand breakage by a 5 min exposure to UV radiation (254 nm), the gel was blotted onto a positively charged nylon membrane (Qiagen) by alkaline transfer [20]. The membrane was hybridised with a digoxigenin-labeled CPN10 probe. Blots were stained as described above.

### Digital imaging

Primary experimental data were digitalised either on a flatbed scanner (Microtek Scanmaker 8700) or on a 35 mm film scanner (NIKON LS-4000). Phosphor imager (Molecular Dynamics) data were imported directly as TIFF images. Images were cropped and optimised for colour saturation using Adobe Photoshop^® ^software. No filters or other image altering functions were employed on the images or parts thereof. Images were combined with vector graphics and text using ClarisDraw^® ^software, version 1.0d.

## Competing interests

The author(s) declare that they have no competing interests.

## Authors' contributions

F.B.Z-V. performed the cloning of CPN10, the pulsed field gel electrophoresis, RT-PCR, the expression of recombinant protein and production of polyclonal antibodies, the construction, transfection and analysis of CPN10::GFP chimera, and the initial immunoblot analyses.

M.K. performed the immune electron microscopy and additional immunoblot experiments.

D.Z. amplified and characterised CPN10 DNA from L. donovani genomic DNA.

J.C. conceived the study, supervised the execution and prepared the final draft of the manuscript.

All authors participated in the drafting of the manuscript, and read and approved the final manuscript.
